# Improving small-scale cultivation of *Spodoptera frugiperda* 9 cells by silanizing glassware

**DOI:** 10.1038/s41598-024-84093-w

**Published:** 2024-12-31

**Authors:** Kristina Worch, Benjamin Ole Mühlnickel, Jana Pieper, Antje Burse

**Affiliations:** 1 Department of Medical Engineering and Biotechnology, Ernst-Abbe-Hochschule, University of Applied Sciences, Carl-Zeiss-Promenade 2, 07745 Jena, Germany; 2 Department of Environmental, Process and Energy Engineering, MCI Management Center Innsbruck, Innsbruck, Austria

**Keywords:** Cell culture, Sf9, Silanization, Reproducibility, Biological techniques, Cell biology

## Abstract

Cultivating insect cells in glass vessels can be challenging. Due to uncontrolled cell adhesion and associated cell loss as well as clumping, the replication of experiments is put at risk. A cost-effective solution to improve and stabilize cultivation may be to silanize glass vessels, making them more hydrophobic and chemically inert. Therefore, the cell growth parameters of *Spodoptera frugiperda* (Sf) 9 cells were characterized in an experiment comparing silanized and non-silanized vessels of three different sizes. Silanization had a significantly positive effect on living cell count and viability in small-scale cultivation (10 ml culture tubes and 50 ml culture flasks) by reducing adhesion of cells to the vessel walls. The treatment also improved the comparability of the biological replicates by reducing the variance of measurements. No such effects could be observed for larger 250 ml culture flasks. Overall, the results indicate that silanization can improve cultivation, especially when working with small glass vessels.

## Introduction

The enormous potential of baculovirus-based expression of heterologous gene products in insect cells has been widely discussed and recognized^1–3^. The cultivation of insect cells, however, can be challenging in terms of reproducibility of viability and cell yield^4^. For instance, insect cells are often cultivated in suspension in conventional glass vessels like culture tubes and flasks, which are available as basic equipment in most cell culture labs. These vessels are rich in hydroxyl groups, forming a hydrophilic surface, to which media and cell components apparently adhere uncontrollably, resulting in a clearly visible cell rim inside the vessel. This can lead to a considerable loss of cells and a reduced living cell count^5^. Especially when working with small volume vessels such as 10 ml culture tubes. As those losses are distributed unevenly over several vessels, an inter-sample and inter-experimental variability of experimental results occurs.

To mitigate the problem, plastic single use shake flasks can be considered^6^, customized media composition^5^ or shear stress reducing additives can be used^7^. However, specialized equipment and media can be expensive, may not be available in the desired size, and the environmental impact of disposable products may argue against their use. A simple and cost-effective alternative to improve reproducibility could be to silanize conventional glass vessels, converting Si–OH bonds to Si–O–Si bonds, thereby forming a durable layer^5,8^. Hence, compared to untreated glass, silanized surfaces are more hydrophobic, more chemically inert and they usually have a lower net charge^8,9^. Due to these advantages, silanizing glass vessels should facilitate the cultivation of insect cells and at the same time cause low costs due to the reusable silanization reagent. In this article, we describe how glass vessels are silanized and present the experimental results investigating the effects of silanization on *Spodoptera frugiperda* (Sf) 9 cell cultivation compared to non-silanization controls at three different scaling levels.

## Methods

### Silanizing glass vessels

**Fig. 1:**
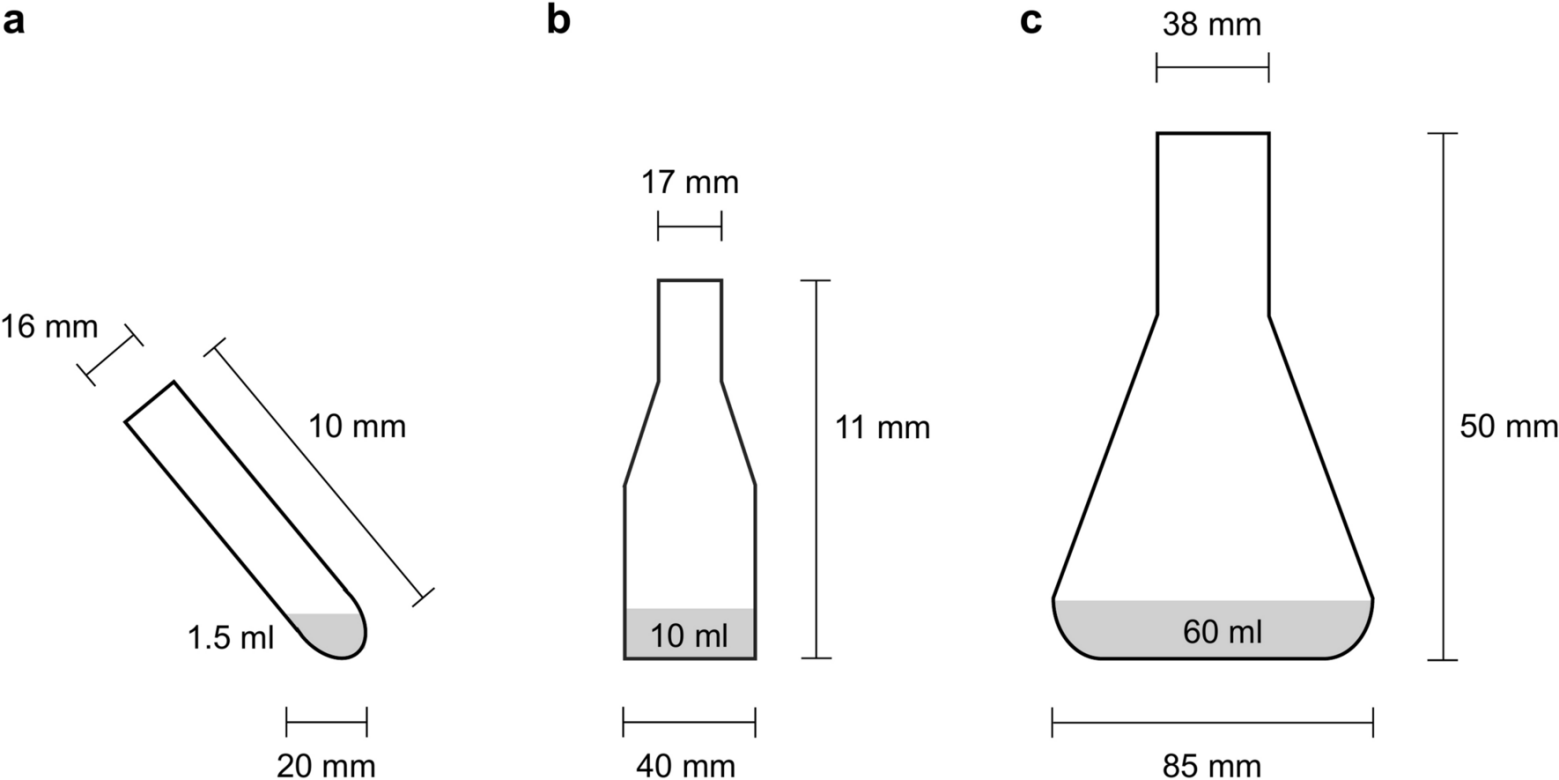
Experimental vessels were (**a**) 10 ml culture tube, (**b**) 50 ml culture flask, and (**c**) 250 ml culture flask.

Clean and dry glassware (Fig. 1) was filled, depending on the cultivation volume, with 3, 20 or 90 ml Sigmacote® (Merck, Darmstadt, Germany) under sterile conditions, respectively. The vessels were swirled for 2 min in such a way that as much surface area as possible was wetted. The silicone oil was removed and stored at 4 °C for further applications. After rinsing six times with distilled water, the glassware was dried at 100 °C for 1 h and finally sterilized at 180 °C for 2 h using a Drying and Heating Chamber (Binder, Tuttlingen, Germany).

### Cell culture and analyses

Sf9 cells (Leibniz Institute DSMZ, Braunschweig, Germany) in the exponential growth phase were seeded at a density of 0.5 × 10^6^ cells/ml in Insect-XPRESS™ medium (Lonza, Köln, Germany). The culture volumes were 1.5 ml in 10 ml culture tubes (Brand, Wertheim, Germany), 10 ml in 50 ml or 60 ml in 250 ml culture flasks (VWR, Darmstadt, Germany). The vessels were covered with ROTILABO® cellulose culture plugs (sizes 13 and 32; Carl Roth, Karlsruhe, Germany) and aluminum foil. Cells were cultivated for 10 days at 27 °C and 180 rpm in a MaxQ™ 6000 shaker with 1.9 cm orbit (Thermo Fisher Scientific, Schwerte, Germany), the 10 ml culture tubes were tilted by 40°. Living cell count and viability were determined by Trypan Blue exclusion using a Countess 3 automated cell counter (Thermo Fisher Scientific, Schwerte, Germany) every 12 to 24 h for up to 11 days. For the 10 ml culture tube and the 50 ml culture flask, 3 biological replicates each were recorded while 2 replicates were measured for the 250 ml culture flask. The Wilcoxon signed rank test with continuity correction comparing the differences against zero, was conducted in R version 4.2.2.

## Results

**Fig. 2:**
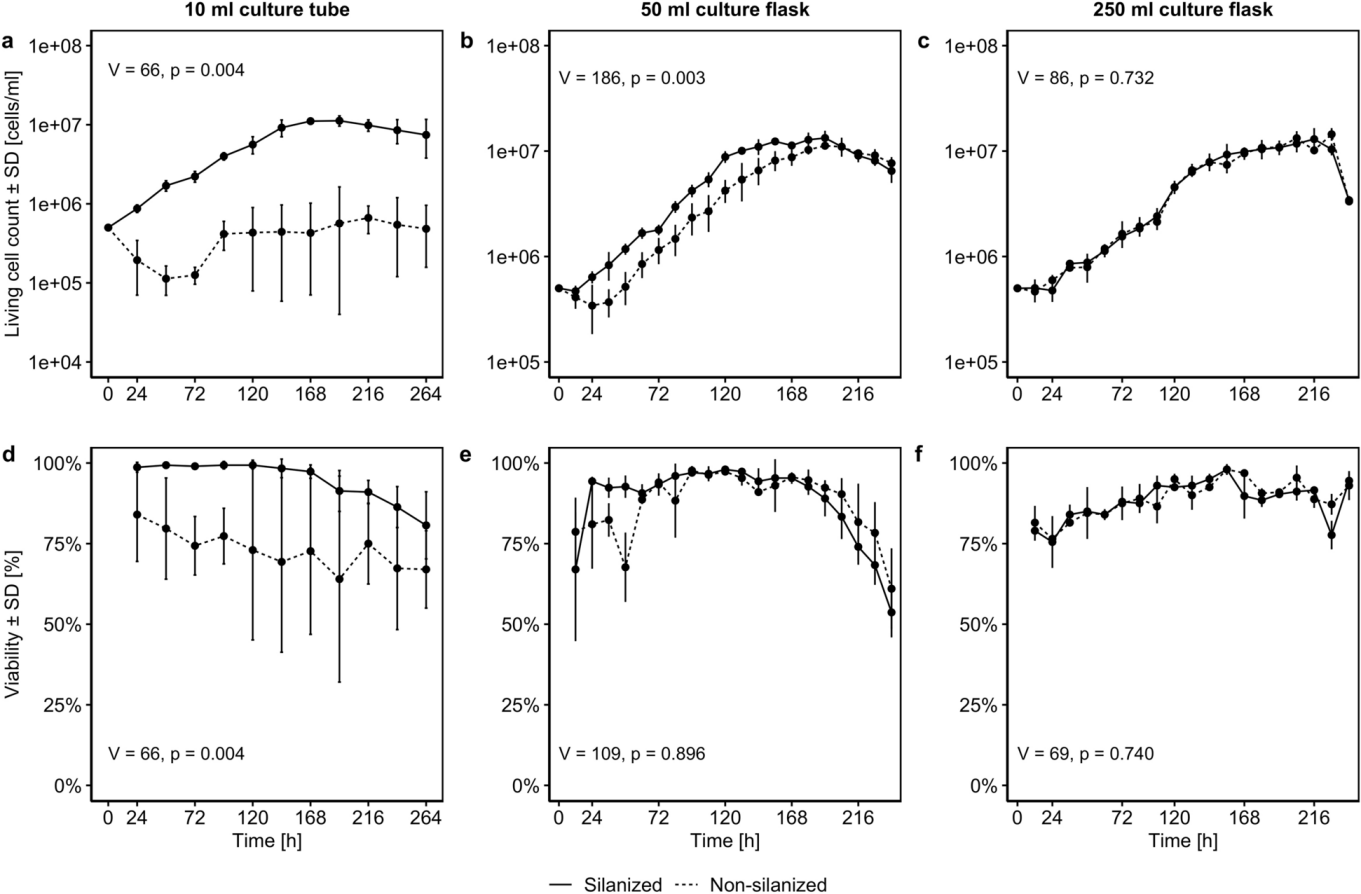
Growth curve (**a**, **b**, **c**) and viability development (**d**, **e**, **f**) of *Spodoptera frugiperda* (Sf) 9 cells in non-silanized and silanized vessels of different sizes. For 10 ml culture tube and 50 ml culture flask, 3 biological replicates each were recorded while 2 replicates were measured for 250 ml culture flask. V and p refer to Wilcoxon signed rank tests comparing the differences between silanized and non-silanized curves against zero.

The cell counts determined were used to generate growth curves and calculate growth parameters for each cultivation vessel (Fig. 2, Table 1). By looking at the growth curves, it is obvious that silanizing considerably improved cultivation at small scale. Compared to non-silanized tubes, in silanized 10 ml tubes a lag phase was not present and cell growth in the exponential phase was tripled, resulting in significantly higher living cell counts and maximum cell density (Fig. 2a) as well as cell viability (Fig. 2d). This observation corresponds with the clearly decreased population doubling time (PDT) and the increased specific growth rate µ shown in Table 1.

**Table 1:** Growth parameters of *Spodoptera frugiperda* (Sf) 9 cells in silanized and non-silanized vessels of different size.

Vessel	Treatment	Lag time [h]	Exponential phase [h]	µ [h^-1^]	PDT [h]
10 ml	Silanized	-14.54 (= 0)	24—168	0.0177	39.117
	Non-silanized	136.83	96—120	0.0059	117.962
50 ml	Silanized	16.10	12—132	0.0262	26.416
	Non-silanized	41.86	48—156	0.0254	27.342
250 ml	Silanized	21.80	24—144	0.0221	31.322
	Non-silanized	20.54	24—144	0.0220	31.478

Parameters were determined according to Murhammer^10^. Note: Lag time: time before exponential phase; µ: specific growth rate; PDT: population doubling time.

To a lesser degree but still significant, silanizing improved cultivation in the 50 ml flasks by decreasing the lag phase by 24 h (Fig. 2b). However, silanizing resulted only in slightly increased viability at the beginning of the cultivation period from 12 to 84 h (Fig. 2e).

For the 250 ml flasks, cultivation did not differ between silanized and non-silanized treatment as cell growth and lag times were comparable (Table 1), and no significant difference could be found for living cell counts (Fig. 2c) as well as for viability (Fig. 2f).

Furthermore, a rim formation of cells and cell debris was observed in all vessels (see supplement). Those in untreated vessels formed on the first day after seeding whereas rims in silanized vessels appeared up to three days later and were smaller in size. Across all vessels, after initially increasing in mass, rims tended to stagnate or decrease by slow reintroduction of cells and debris into the suspension. As indicated by the smaller standard deviations in Fig. 2, silanizing also reduced the variance of living cell count and viability, clearly improving the comparability of biological replicates.

## Discussion

Silanizing glassware reduced adhesion of cells and cell debris to the vessel walls visible as rim formation especially at the beginning of cultivation. As more cells remained in suspension, cell growth was improved accordingly. The benefits of silanizing seemed to depend on the ratio between the cell suspension volume and the diameter of the cultivation vessel. Silanizing had a positive, reproducible effect on the living cell count and viability in small-scale cultivation formats (10 ml culture tubes and 50 ml culture flasks), but not when working with 250 ml culture flasks. However, silanizing may still be beneficial for larger vessels because it can reduce cleaning efforts as less cells or other organic material stick to the vessel surface.

Silanizing culture vessels is easy and affordable. The modification is durable, allowing researchers to use silanized vessels for many experiments. In addition, the coating can easily be renewed if there are signs of wear^11^.

Reproducible and low-cost cultivation of Sf9 cells on a small scale can be particularly useful for the incubation of cells after cryopreservation or for the transfection of suspension cells with baculoviral DNA^12,13^. However, since the vessel wall is altered, its interaction with media components and other substances such as transfection reagents or viral particles may also be affected, and the impact of silanization should be pretested for other applications.

## Conclusion

While previous research assumed that silanizing glass vessels might reduce cell adhesion and enhance cell growth, our data indicate that it does and that it is most useful when working with Sf9 cells in small culture glass vessels. It improves the reproducibility of experiments and can be considered a simple and cost-effective alternative to plastic single use culture wear and additives that reduce adhesion.

## Supplementary Information

Supplementary Information.

## Data Availability

Raw data and the analysis script are available at http://dx.doi.org/10.17605/OSF.IO/W8M2C
