# Supplementary material for: Improving small-scale cultivation of Spodoptera frugiperda 9 cells by silanizing glassware
Source: Sci Rep. 2024 Dec 31;14:32172. doi: 10.1038/s41598-024-84093-w (PMC11688440; doi:10.1038/s41598-024-84093-w)
Supplement: Supplementary file 1 — Supplementary Information. [file 41598_2024_84093_MOESM1_ESM.docx]

**Supplement**

**Figure S1:** Rim formation in silanized (left) and non-silanized (right) 10 ml culture tubes over a period of 10 days, as indicated by the numbers on the pictures left hand side.

**Figure S2:** Rim formation in silanized (left) and non-silanized (right) 50 ml culture flasks over a period of 10 days, as indicated by the numbers on the pictures left hand side.
